# Traffic noise reduces foraging efficiency in wild owls

**DOI:** 10.1038/srep30602

**Published:** 2016-08-18

**Authors:** Masayuki Senzaki, Yuichi Yamaura, Clinton D. Francis, Futoshi Nakamura

**Affiliations:** 1 Graduate School of Agriculture, Hokkaido University, Kita 9, Nishi 9, Kita-Ku, Sapporo, 060-8589, Japan; 2 Department of Forest Vegetation, Forestry and Forest Products Research Institute, 1 Matsunosato, Tsukuba, Ibaraki, 305-8687, Japan; 3 Department of Biological Sciences, California Polytechnic State University, San Luis Obispo, CA 93407, USA

## Abstract

Anthropogenic noise has been increasing globally. Laboratory experiments suggest that noise disrupts foraging behavior across a range of species, but to reveal the full impacts of noise, we must examine the impacts of noise on foraging behavior among species in the wild. Owls are widespread nocturnal top predators and use prey rustling sounds for localizing prey when hunting. We conducted field experiments to examine the effect of traffic noise on owls’ ability to detect prey. Results suggest that foraging efficiency declines with increasing traffic noise levels due to acoustic masking and/or distraction and aversion to traffic noise. Moreover, we estimate that effects of traffic noise on owls’ ability to detect prey reach >120 m from a road, which is larger than the distance estimated from captive studies with bats. Our study provides the first evidence that noise reduces foraging efficiency in wild animals, and highlights the possible pervasive impacts of noise.

Anthropogenic noise (hereafter “noise”) is increasing globally and mounting evidence suggests that noise can negatively affect wild animals in many ways^1^,^2^,^3^. Of these impacts, masking from noise, where it interferes with an organism’s ability to detect or discriminate biologically relevant signals, appears to be especially problematic^4^,^5^,^6^. Although several studies have examined impacts of masking using “quiet *versus* loud designs”, to fully understand and reduce the severity of masking, quantifying wildlife responses to a range of noise exposure levels is critical^5^,^6^,^7^.

Compromised foraging efficiency in animals, especially in acoustic predators such as owls and bats, is among the main concerns regarding impacts of novel acoustic environments created by noise^8^,^9^,^10^. This is because declines in foraging efficiency likely influence their distributions by altering behavior and reducing habitat suitability^11^,^12^ and thereby may alter predator-prey interactions that have ecosystem-wide consequences^13^. Nevertheless, only a few laboratory experiments with limited sample sizes have examined noise impacts on foraging efficiency, and only in two bat species^8^,^11^,^12^ and in a single owl species^14^. Thus, to clarify whether negative effects of noise on foraging efficiency in acoustic predators are widespread, we must understand the degree to which noise degrades foraging efficiency in acoustic predators in the wild^5^,^6^.

The objective of this study was to experimentally determine the relationship between foraging efficiency of wild acoustic predators and noise levels common to many landscapes. We studied nocturnal owls because they are specialized acoustic predators, have cosmopolitan distributions, and have different audible ranges and hunting techniques than bats. We conducted novel field playback experiments using two types of sounds, traffic noise (hereafter “TN”) and artificial prey rustling sound (hereafter “APRS”) (Fig. 1). Playback of TN allowed us to isolate effects of noise from other confounding factors, such as habitat changes, visual disturbance of moving vehicles and lights, etc^15^,^16^,^17^. Because owls localize and attack prey using prey-generated rustling sounds at frequencies spanning 6–8.5 kHz^18^, we digitally developed APRS (Fig. 2a) and found that owls in the wild are attracted to playback of these sounds (see supplementary Fig. S1), providing a method for quantifying prey detection among wild owls under a variety of acoustical conditions. In field experiments, we played back APRS at constant amplitude under various TN exposure levels at many locations in northern Japan, and thereby examined the effect of TN on owls’ ability to detect APRS. Finally, we estimated the compromised foraging range by noise near roads. To the best of our knowledge, this is the first study to examine effects of different levels of TN on foraging efficiency in acoustic predators in the wild.

## Results

We conducted 367 playback experiments in northern Japan (see supplementary Fig. S2), and recorded a total of 92 owls in 76 playback experiments (Table 1). After exclusion of owls that did not satisfy our analytical criteria (*n* = 14, see “Materials and Methods”), we analyzed 78 owls in 63 playback experiments (45 short-eared owls *Asio flammeus* and 33 long-eared owls *Asio otus*). The best model included sound pressure level (SPL) of TN and suggested owls’ ability to detect prey was negatively associated with SPL of TN (Table 2, Fig. 3a). Species ID and the interaction term had no association with owls’ ability to detect prey (Table 2).

In addition, to estimate relationship between road distances and owls’ ability to detect prey, we also measured SPL of TN at various distances from road. Result of model selection showed SPL of TN attenuated quadratically with distance from road (Table 2, Fig. 3b), indicating impacts of traffic noise on owls’ ability to detect prey has the potential to reach >120 m from a road (Fig. 3b). In other words, owls’ ability to detect prey was impacted even at the lowest level of TN (40 dB[A]) and was approximately 17% lower than detections in ambient sound conditions (Fig. 3a).

## Discussion

Using a novel field-based experimental approach, we show that owls’ ability to detect prey is negatively impacted by increases in TN. Masking of the signal occurs when there is spectral and temporal overlap between the signal and noise^5^. Because APRS experience considerable spectral overlap by TN (Fig. 2a,b), reduction of owls’ ability to detect prey could be caused by increasing acoustic masking with increasing amplitude of TN^11^,^12^, although whether the signal was masked depends on the acoustic processing abilities of the owls and how they hear sound in noise. Additionally, it is not mutually exclusive that distraction and/or avoidance to TN play some role for explaining decreases in prey detection. For example, previous works with bats suggests that distraction/avoidance to noise had larger impact on bats’ ability to detect prey than masking^8^.

Owls’ ability to detect prey was impacted even at the lowest level of TN (40 dB[A]) and was approximately 17% lower than that of ambient conditions (Fig. 3a). This corresponds to a distance of 120 m estimated from our model predicting noise levels from distance to the road and is twice the distance estimated for impacts on bats’ prey detectability due to TN^9^. Methodological differences between laboratory and field studies could explain these differences. For example, laboratory experiments conducted in a restricted area could overestimate prey detectability of acoustic predators because in a confined laboratory setting they circle above the experimental foraging area in flight and may have more chances to detect their prey. In contrast, in the field acoustic predators typically forage in linear flight and would have fewer opportunities to detect prey sounds^11^. Alternatively, such difference may be, at least partially, due to differences of audible range and/or sensitivity to sounds between birds and bats (i.e., owls cannot detect sounds at frequencies above 15 kHz^18^ while echolocating bats use sounds at frequencies up to 120 kHz)^11^ and differences in prey-generated rustling sounds between the experiments.

Masking of real or artificial prey rustling sounds by traffic noise should invariably reduce foraging efficiency to some degree. However, hunting owls may be able to take advantage of directional masking release where rustling sounds and background noise propagate from different directions. Distraction, in which owls attend to traffic noise rather than rustling sounds, could also explain declines in prey detectability and could operate along side masking. However, it is also possible that distraction or compromised attention could decrease with habituation to traffic noise over time. Distinguishing among these potential mechanisms must be a next step. Additionally, it is also critical to understand whether declines in prey detection scale to responses most relevant to population persistence, such as site abandonment or impact actual foraging success, body condition and reproductive success of animals occupying noisy areas^11^,^19^,^20^.

In addition, there are several differences between this study and natural conditions. First, omnidirectional TN used here differs from horizontal TN propagation from roadways that wild owls encounter in nature. Thus, future work evaluating how directional masking release changes detection of APRS is needed. Second, we used a representative TN sound recording in the experiments based on comparisons among several TN sounds. Although this isolates noise amplitude as a single factor that varied among treatment levels, it also does not reflect TN variation due to variable traffic speeds, densities and environmental conditions, indicating that future work should focus on how possible TN variation affects owls’ ability to detect prey. Moreover, high frequency components of TN attenuate faster with distance from roads than lower frequency components, suggesting overestimation of the masking effects of TN playbacks at amplitudes reflective of 55, 105, 155, 205 m from the road. However, because APRS playbacks were louder than natural prey rustling sounds and APRS might be easier for owls to detect than actual prey rustling sounds with broadband energy, effects of TN on owls’ prey detection may extend well-beyond our 120 m estimate.

Despite the need to parse the effects of how directional masking release, real versus artificial prey sounds and high frequency components simultaneously contribute to estimated impacts with respect to distance from roads, we provide the first evidence that noise reduces foraging efficiency in a wild predator in a natural situation. Additionally, our analysis of sound level attenuation with distance from the road suggests that declines in prey detection occur at distances twice that estimated for bats from lab studies^11^, at least in our study region. Nevertheless, given our playback is representative of traffic noise propagating from other roadways (see supplementary Fig. S3), it is likely that impairment of foraging at similar distances is generalizable to other roadways. Moreover, a recently published captive study showed that experimental playback of compressor noise, which has similar power spectrum with traffic noise, negatively impacts hunting behavior of northern saw-whet owls (*Aegolius acadius*) at sound levels as low as 46 dB(A), which corresponds to approximately 800 m from compressor stations^14^. These potentially sizable footprints from energy-sector and traffic noise highlight the pervasive impacts of noise on acoustic predators because many sources of noise, including road densities, are high and increasing^4^. For example, 83% of the continental US is within 1061 m of a road^21^, and globally, >25 million kilometers of new roads are anticipated by 2050^22^. Key to fully understanding noise-impacts on acoustic predators will require knowledge of how the magnitude of noise-impacts varies depending on road densities, arrangements and traffic volumes and speeds. Moreover, it is critical to understand how common prey species respond to roadways and traffic and determine whether the cumulative effects are additive, synergistic or even antagonistic, as some nocturnal small mammals appear to increase in noise exposed areas^23^ and along roadways^24^. Regardless of the shape of these interactions, it is likely that wild owls and other acoustically-oriented predators will continue to be impacted by noise.

## Methods

### (a) Preparation of the traffic noise for playback experiments

Vehicle noise was recorded at the prefectural road #1046 in Yufutsu plain, central Hokkaido, late December 2014. The recording was conducted between 22:00 to 02:00 on a clear day when wind speeds were less than 1 m/s. We set a recorder (PCM-D100, Sony Corporation, Tokyo, Japan; frequency response ± 2 dB between 20 Hz and 45 kHz) with a sound pressure meter (Sound Level Meter TYPE 6236, ACO CO., LTD, Miyazaki, Japan) at a height of 1.5 m and 5 m distance from the road. Then, for each of 20 passing vehicles at constant speed (60 km/h), we recorded its noise and measured its sound pressure level (SPL) as the A-weighted equivalent continuous noise level during five seconds at nearest distance to a vehicle (*L*_*eq*_ [5 s], fast response time, re. 20 *μ*Pa, A-weighting). For these, we used the A-weighted filter because this filter provides better measurement of acoustic energy relevant to birds at frequencies between 1.0 and 9.5 kHz^25^, which cover entire frequency range used by hunting owls^18^. Finally, we created a 1 min exemplar of TN sound consisting of 12 vehicle pass-by events, which contained energy up to 40 kHz, but had the most energy below 10 kHz (Fig. 2b). This traffic level was found along roads in many national parks, national forests, and protected areas globally^19^. Although it is better to use different TN sounds in each playback experiment to capture potential heterogeneity in traffic noise present in different locations or times, because our primary interest is to quantify effect of amplitude alone on owls’ ability to detect prey, we used this single TN sound file in all playback experiments based on comparisons of frequency spectra among several TN sounds recorded at different locations (see supplementary Fig. S3). We also created a 1 min control sound file that had no acoustic energy. In addition, to understand how sound levels attenuate with distance from the roadway, for each of 20 passing vehicles at known speed (i.e., 60 km/h), we measured its sound pressure level (SPL) as the A-weighted equivalent continuous noise level during five seconds at nearest distance to a vehicle (e.g., LAeq [5 s]) at 5, 55, 105, 155, and 205 m from the road.

### (b) Preparation of the artificial prey rustling sound for playback experiments

When small-mammals walk on the ground, they produce rustling sounds which are short and contain a wide range of frequencies^18^. Owls can precisely locate these rustling sounds, especially at frequencies between 6 and 8.5 kHz^18^. Because they respond strongly to stimuli at these frequencies, we created sound files consisting of an upsweeping element of 0.4 s in duration spanning 3.0–9.0 kHz separated by 0.1 s (sampling rate: 192 kHz). For each file, the elements were repeated eight times, followed by 6 s with no acoustic energy (Fig. 2a). This 10 s section was then repeated six times to create a one-minute artificial prey rustling sound, which is similar in structure to rustling sounds made by actual prey^18^. All sound analyses and clip generation were conducted in Sound Forge Audio Studio 10.0 (Sony, Tokyo, Japan).

### (c) Study area and field playback experiments

To make certain that we could obtain sufficient sample sizes, we selected two study areas in northern Japan where many owls overwinter. Specifically, field experiments were conducted in Yufutsu plain, central Hokkaido and in Sendai plain, northern Honshu (see Supplementary Fig. S2). Both landscapes are predominantly agricultural fields and semi-natural grasslands (see Supplementary Fig. S2), providing suitable environments for our target study species. We established 103 playback experimental plots in these two areas (45 in Yufutsu plain and 58 in Sendai plain, northern Honshu) (see Supplementary Fig. S2). In the study area, an individual short-eared owl territory size was estimated to be approximately 5 ha (M. Senzaki, personal observations), which nearly equals an area with 130 m radius. Thus, to prevent double sampling, adjacent plots were spaced by >500 m. In addition, we did not establish plots in areas with tall trees or streetlights to prevent potential effects of these factors on sound propagation or owls’ behaviors respectively. Playback experiments were conducted at least once in each plot between 1700–0500 h on both clear and cloudy nights, when wind speeds were <2 m/s, from December 2014 to March 2015, which corresponded with owls’ wintering periods. The average number of playback experiments at each plot (±SD) was 3.56 ± 1.23. When owls were sampled in a plot, we did not conduct any additional playback experiments in the same plot three or more days to minimize effects of habituation.

**Figure 1:**
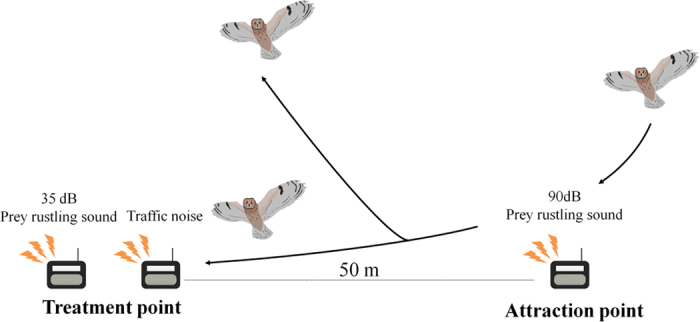
Schematic of the playback experimental set up.

**Figure 2:**
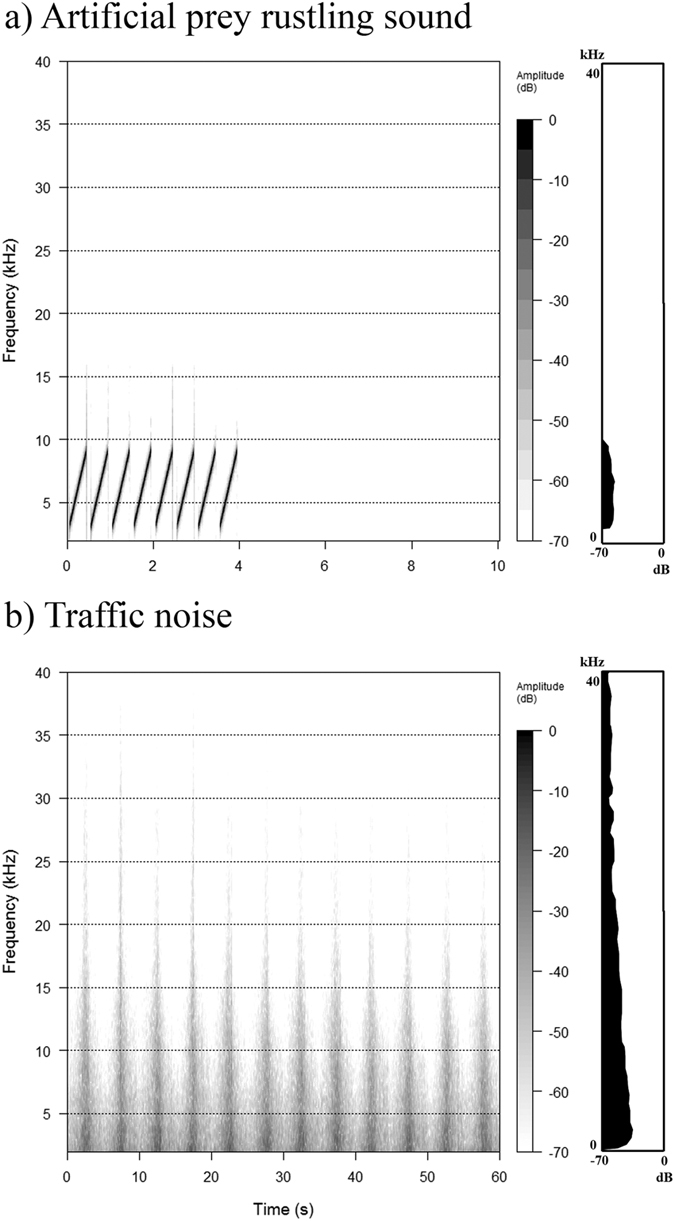
Spectral characters, relative amplitudes (left panel) and power spectra (right panel) of (**a**) ARPS and (**b**) TN.

**Figure 3:**
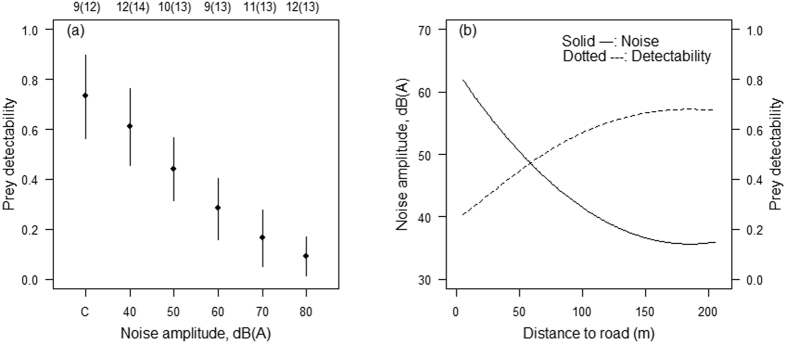
(**a**) Estimated owls’ ability to detect prey under noise exposure levels (with 95% CI). “C” indicates control experiments. Detectability at C is estimated using average background sound level (32 dB). Top figures indicate number of experiments (number of owls analyzed). (**b**) Relationships between road distances and noise levels and owls’ prey detectability. The owls’ ability to detect prey was estimated based on linear regression equation presented in (**a**).

A plot consisted of an attraction and treatment point spaced 50 m apart (Fig. 1) with one and two speakers (PDX-B11: Yamaha, Hamamatsu, Japan; frequency response ± 10 dB between 55–20 kHz) connected with players (WALKMAN NW-E080, Sony Corporation, Tokyo, Japan), respectively. Although traffic noise propagates horizontally across the landscape, and mimicking directional propagation can be carefully controlled in laboratory conditions^11^,^12^, we set all speakers on the ground facing upwards to ensure omnidirectional propagation of attraction and treatment point sounds across the landscape. This ensures fairly equal amplitudes of playback sounds in all directions, which was important when owls could approach from any direction. On nights when background SPL ≤ 35 dB(LAeq[1 min]), we broadcasted TN or the silent sound file with no acoustic energy (hereafter “control sound”) from one speaker at the treatment point until the end of the experiment. Amplitude of TN was randomly chosen to be approximately 40, 50, 60, 70, or 80 dB(LAeq [5 s]) at 1.5 m height above the speaker, representing sound levels measured at different distances from a roadway. After 1 min of TN broadcast at the treatment point, we first broadcast APRS at 90 max dB(A) at a height of 1.5 m above the attraction point speaker for 1 min to attract owls from the larger surrounding area. When the playback was finished, we immediately broadcast APRS at 35 max dB(A) at a height of 1.5 m above the second speaker at the treatment point for 1 min. Although 35 dB(A) is louder than natural prey sounds^11^,^12^, we used the value to ensure that owls at attraction points could detect APRS at treatment points at least under control playback conditions. We tracked owls attracted to the attraction point and determined whether they could subsequently detect APRS at the treatment point. Owls that actively entered the range within 10 m from the attraction/treatment point (e.g., owls hovering and/or flying circular over the speaker) were determined to detect APRS in each point. When we observed attacks and/or chases between attracted owls and/or when we could not determine whether owls in attraction points were detecting APRS in treatment points because they landed on the ground, they were not included in subsequent analyses. We also excluded experiments with no owls detected from any analysis. Because flying owls could be observed at approximately 50 m distance from an observer, observations were conducted 30 m from both attraction and treatment points using a night scope (ATN Night Spirit XT, California, USA) and binoculars (MONARCH 8 × 42, NIKON CORPORATION, Tokyo, Japan).

### (d) Data analysis

We used Generalized Linear Model (GLM) with Gaussian error to examine how TN decreases with distance from a road. We treated SPL as a response variable, and distance from a road (m) and its quadratic term (m^2^) as explanatory variables.

We examined effects of TN on owl’s ability to detect prey using Generalized Linear Mixed Model (GLMM) with Binomial error. We treated whether owls detected APRS at the treatment point as the response variable, SPL of TN, or ambient SPL measured prior to the start of control trials, species ID (long- or short-eared owl) and the interaction of these variables as explanatory variables. Plot ID and Study region (Yufutsu or Sendai) were treated as random variables. Although identifying whether the same individuals were recorded within a specific plot was difficult due to low light levels, treating plot ID as a random effect can account for possible repeated sampling of the same individuals. For experiments with control sound, SPL measured before the start of the experiments was used. We constructed models for the combinations of all possible covariates, ranked them by Akaike’s information criterion for small sample size (AICc), and considered covariates in the best model as meaningful predictors. These analyses were conducted using “lme4” (v. 1.1–5)^26^ and “MuMIn”(v. 1.9.13)^27^ with R software (v. 2.15.3)^28^.

### (e) Ethical statement

All experiments were performed in accordance with relevant guidelines and regulations. All experimental protocols were approved by the Japanese Ministry of the Environment.

## Additional Information

**How to cite this article**: Senzaki, M. *et al*. Traffic noise reduces foraging efficiency in wild owls. *Sci. Rep*. **6**, 30602; doi: 10.1038/srep30602 (2016).

## Supplementary Material

Supplementary Information

## Figures and Tables

**Table 1:** Summary of field experiments.

	Yufutsu	Sendai	Total
Number of study plots	45	58	103
Number of experiments	210	157	367
Number of owls	21	71	92
long-eared owl	7	29	36
short-eared owl	14	39	53
ural owl	0	3	3

**Table 2:** Results of GLM examining how TN decreases with distance from a road and GLMM examining effects of TN on owl’s ability to detect prey.

Variables	Model rank				*β*	SE
	1	2	3	4		
Traffic noise level						
Distance from road	+	+			−0.30	0.01
Distance from road^2^	+		+		0.00	0.00
df	4	3	3	2		
ΔAICc	0.00	115.80	199.44	295.71		
Weight	1.00	0.00	0.00	0.00		
Owls’ prey detectability						
Trafic noise	+	+	+		−0.07	0.02
Species_ID		+	+			
TN X SP_ID			+			
df	4	5	6	3		
ΔAICc	0.00	2.28	4.60	17.07		
Weight	0.70	0.23	0.07	0.00		

For GLM, we treated SPL as a response variable, and distance from a road (m) and its quadratic term as explanatory variables. For GLMM, we treated whether owls detected APRS at the treatment point as the response variable, SPL of TN, species ID and interaction of these variables as explanatory variables and plot ID and Study region (Yufutsu or Sendai) as random variables. Variables included in models are indicated with plus sign. “TN X SP_ID” indicates the interaction term between traffic noise and species ID and “Weight” refers to Akaike Weights. Parameter estimates (*β*) and its standard errors (SE) in the best models are also given.
