# Supplementary material for: Traffic noise reduces foraging efficiency in wild owls
Source: Sci Rep. 2016 Aug 18;6:30602. doi: 10.1038/srep30602 (PMC4989872; doi:10.1038/srep30602)
Supplement: Supplementary Information [file srep30602-s1.pdf]

Supplementary figures

Traffic noise reduces foraging efficiency in wild owls

Masayuki Senzaki, Yuichi Yamaura, Clinton D. Francis, Futoshi Nakamura

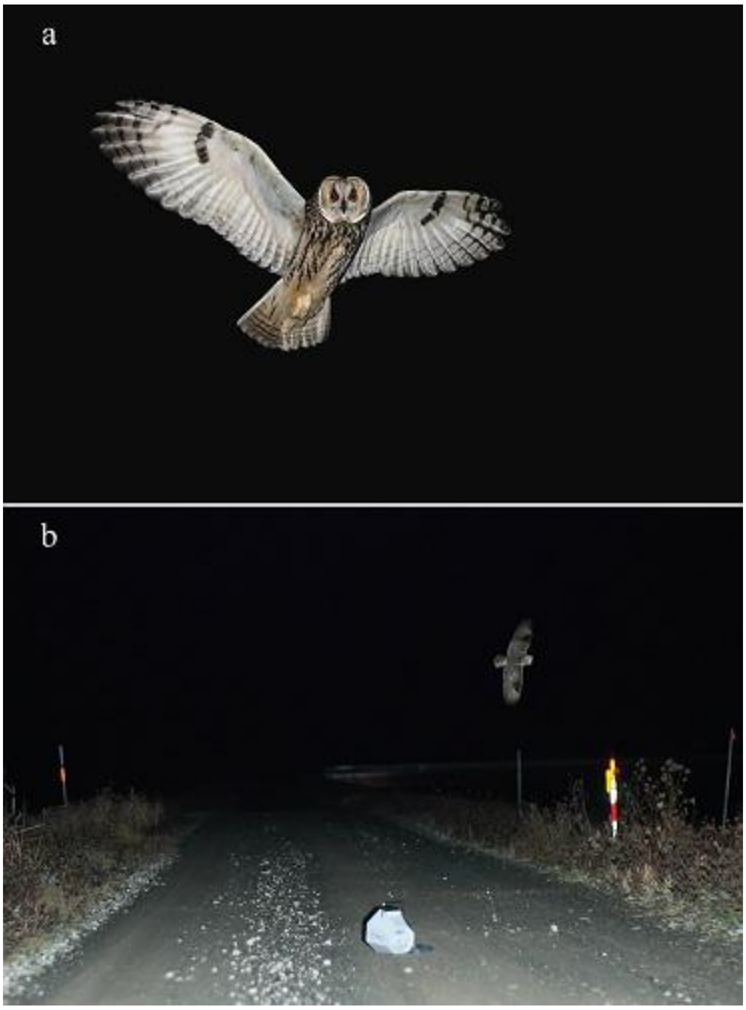

Figure S1.
(a) Long-eared (b) and short-eared owls attracted by artificial prey rustling sound. The speaker at lower centre of (b) was producing artificial prey rustling sound. Photographs were taken in preliminary survey periods.

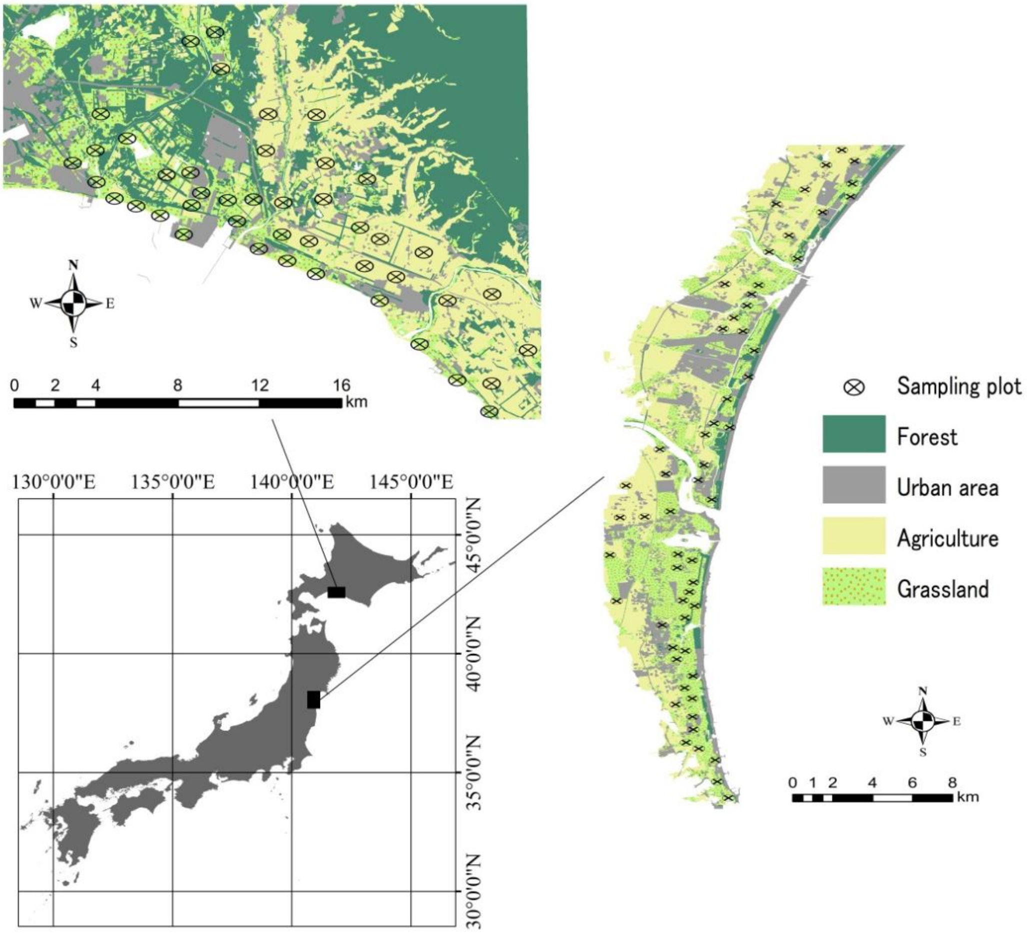

Figure S2.

Map of the study areas. Top and right panels are Yufutsu and Sendai plains. The map was created by modifying 1:50000-scale actual vegetation map in the fifth Japanese National Survey of the Natural Environment conducted by the Japanese Ministry of the Environment (downloaded from http://gis.biodic.go.jp/webgis/sc-023.html) with ArcGIS, [10.0], (http://www.esri.com/software/arcgis/arcgis-for-desktop).

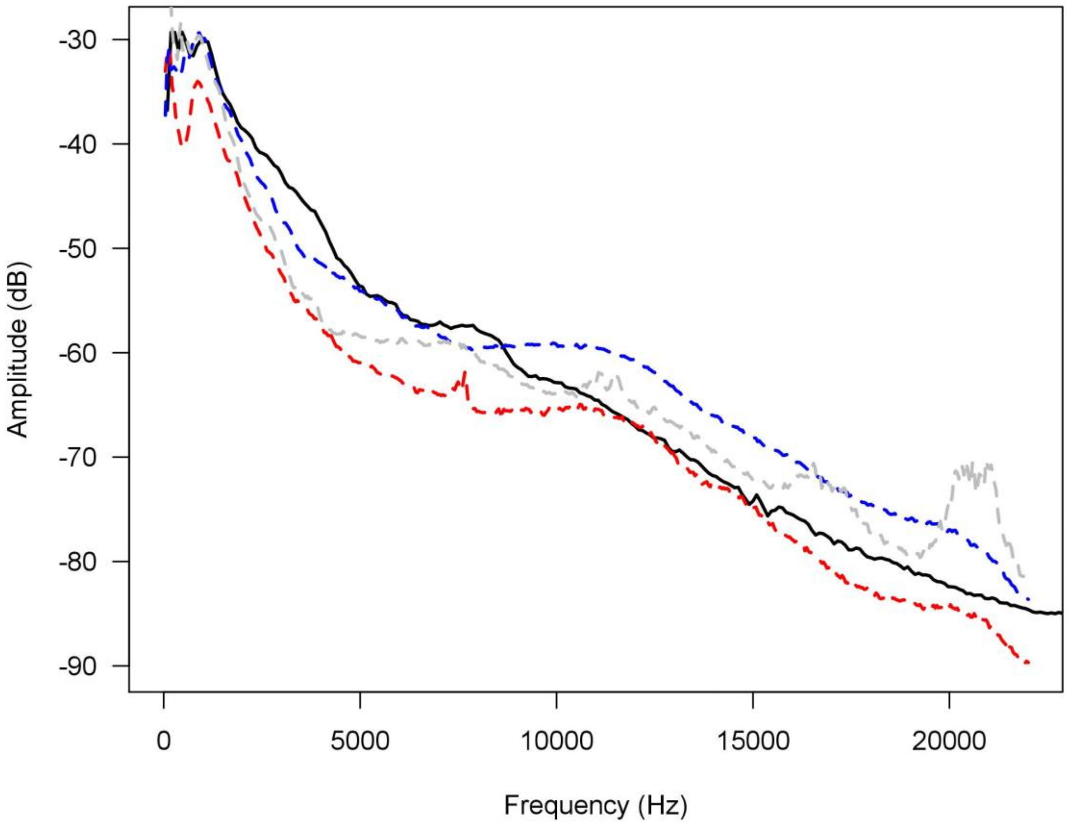

Figure S3.

Black line indicates the power spectrum of traffic noise file used in the experiments, and other three colored dashed lines are power spectra recorded from around San Luis Obispo, California, USA.
